# Case Report: Application of extracorporeal shockwave therapy in medial epicondylitis with concomitant ulnar nerve instability: a case series with long-term follow-up

**DOI:** 10.3389/fresc.2026.1677404

**Published:** 2026-03-02

**Authors:** Larisa Ryskalin, Federica Fulceri, Francesco Busoni, Elisa Colomo, Paola Soldani, Marco Gesi

**Affiliations:** 1 Department of Translational Research and New Technologies in Medicine and Surgery, University of Pisa, Pisa, Italy; 2 Studio Radiologico Busoni, Private Practice, Pisa, Italy

**Keywords:** combined shockwave therapy, disability, elbow tendinopathy, medial elbow pain, ulnar nerve, upper extremity

## Abstract

**Background:**

Medial epicondylitis is an overuse syndrome characterized by degeneration of the flexor-pronator tendons in the elbow, resulting from repetitive forced wrist flexion and forearm pronation. Due to its anatomical location, medial epicondylitis patients may also feature ulnar nerve instability, which can exacerbate symptoms and negatively impact treatment outcomes. Although conservative treatments remain the cornerstone of care for managing medial epicondylitis, the optimal treatment method remains an open question.

**Objective:**

To evaluate the effects of a combined extracorporeal shockwave therapy (ESWT) protocol on pain, symptom severity, and functional outcomes in medial epicondylitis patients with concomitant ulnar nerve instability.

**Design:**

Retrospective case series study with two-year post-treatment follow-up.

**Setting:**

Center for Rehabilitative Medicine “Sport and Anatomy”, University of Pisa.

**Interventions:**

Patients underwent a combined ESWT using the Duolith SD1 ultra device (Storz Medical AG., Switzerland), consisting of sequential focal (0.15–0.20 mJ/mm^2^, 5–6 Hz, 1,000 shocks) and radial (1.3–1.8 mJ/mm^2^, 14 Hz, 2,000 shocks) shockwave application per session. Each patient received three to five weekly sessions.

**Participants:**

Medial epicondylitis patients with concomitant ulnar nerve involvement who underwent a combined ESWT protocol between September 2019 and May 2023.

**Main outcome measures:**

Pain severity and upper limb disability were assessed with the numerical rating scale, the shortened Disabilities of the Arm, Shoulder and Hand questionnaire, and the Ulnar Neuropathy at the Elbow Questionnaire. Patient treatment satisfaction was evaluated with the Roles and Maudsley score.

**Results:**

Of the reviewed 15 consecutive medical charts, only three subjects fulfilled the inclusion criteria. Two patients showed a marked decrease in pain and improved functionality scores at all time points; one patient remained unchanged throughout the study; no adverse effects were observed.

**Conclusions:**

This retrospective study suggests that ESWT may be efficacious and safe for treating medial epicondylitis patients with concurrent ulnar nerve instability. Prospective studies with a larger sample size are needed to warrant the present results.

## 1 Introduction

**Figure 1:**
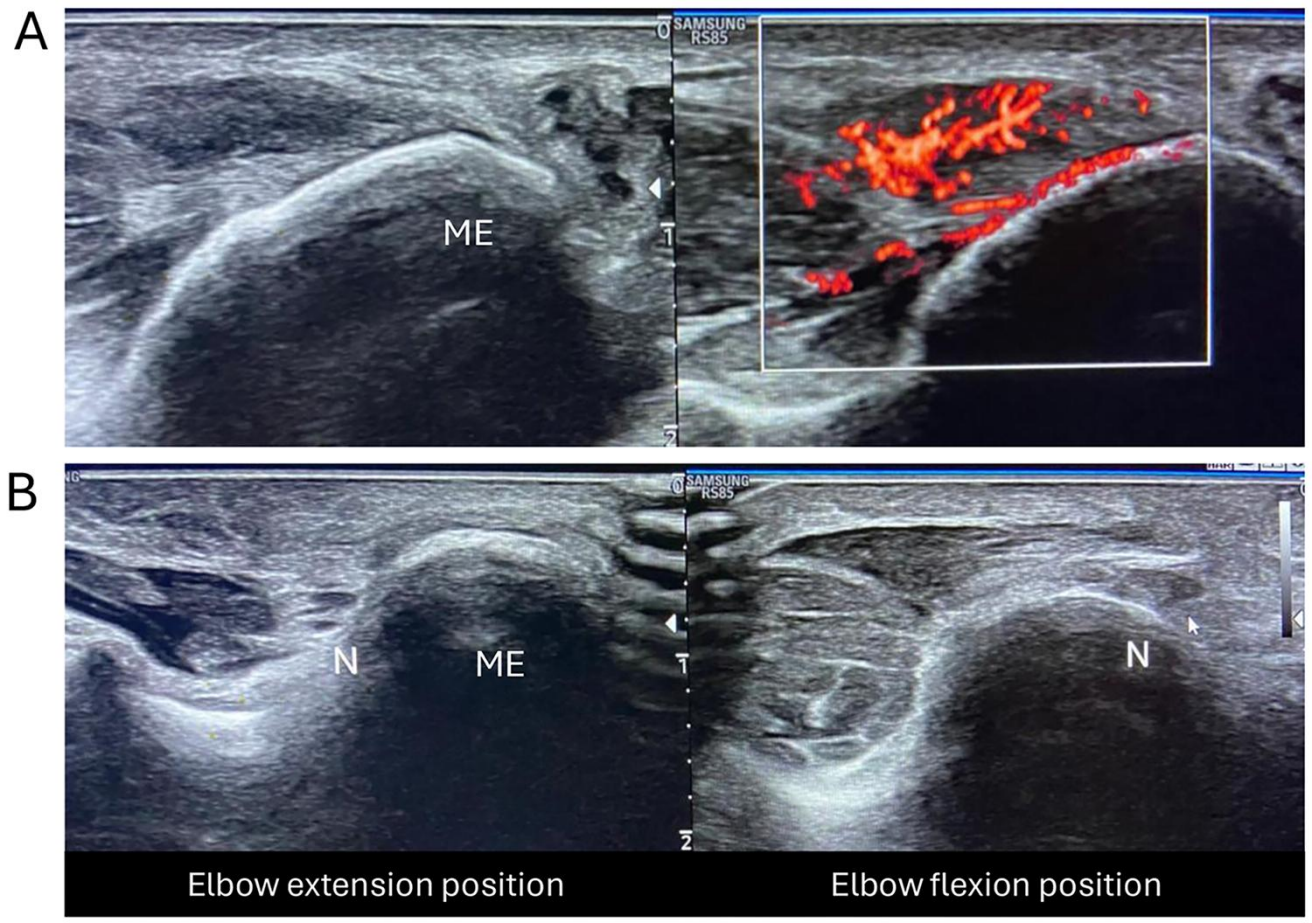
Ultrasound imaging of the medial elbow. **(A)** Longitudinal view and Color Doppler showing FPG common tendon entesopathy and tendinosis. **(B)** US axial view of ulnar nerve **(N)** displacement over the medial epicondyle **(ME)** during elbow flexion.

Commonly referred to as “golfer's elbow”, medial epicondylitis (medial elbow tendinopathy) refers to pathological changes in the musculotendinous origin of the flexor-pronator muscle group at the humeral medial epicondyle (1, 2). The majority of the literature on medial epicondylitis suggests that the degenerative changes in the common flexor-pronator tendon are due to chronic, repetitive forced wrist extension and forearm supination (2, 3) (Figure 1A). Medial epicondylitis typically affects the middle-aged population, and it is commonly ascribed to overload/overhand throwing sports, as well as manual jobs that involve similar repetitive motions and biomechanical demands (3–6). Compared to lateral epicondylitis, medial epicondylitis occurs 5–10 times less frequently, accounting for a prevalence of <1% in the general population (1, 3, 7). As a result, clinical data and literature on medial epicondylitis are quite limited. Patients with medial epicondylitis often complain of progressive-onset and insidious medial-sided elbow pain without known antecedent trauma, swelling, and tenderness that are exacerbated with activity, especially those requiring forearm pronation/wrist flexion.

Due to the close anatomical relationship between the common flexor-pronator tendon and the ulnar nerve, which is located posterior to the medial epicondyle, ulnar nerve pathology is a common comorbidity in medial epicondylitis patients, reported in up to 60% of cases (2, 3, 7–10). Ulnar nerve irritation may stem from the spread of local inflammation and/or direct compression from inflamed tendinous tissue, chronic mechanical stress, and/or dynamic nerve instability consequent to alterations in Osborne's ligament, leading to unstable ulnar nerve positioning (2–5, 11, 12). In such cases, the ulnar nerve may transiently displace medially over the medial epicondyle during elbow flexion, returning to its original position with extension (8). This dynamic subluxation may lead to increased nerve irritation, local swelling and tenderness, establishing a vicious cycle of progressive tendinopathy and ulnar nerve irritation that further exacerbates pain and functional impairment.

At present, conservative management with nonoperative approaches remains the mainstay of treatment, including activity modification, non-steroidal anti-inflammatory medications, corticosteroid injections, electrical stimulation, splint and bracing (7, 13). However, there is a paucity of literature regarding medial epicondylitis conservative management, probably also due to its infrequent incidence of only approximately 10% of all epicondylitis diagnoses. Again, most treatment approaches lack strong evidence, making it difficult to define the optimal treatment modality (14). Within this frame, extracorporeal shockwave therapy (ESWT) has gained attention in the field of orthopedics and traumatology for the treatment of several musculoskeletal disorders, showing promising outcomes in pain relief and functional recovery (15–20). Increasing evidence indicates that ESWT may sort various beneficial effects on numerous bone and soft-tissue pathologies, way beyond the mere mechanical disintegrative effect, as generally assumed (21–27). When looking at soft tissue diseases of the upper limb, ESWT was shown to effectively relieve pain and improve function in patients with rotator cuff tendonitis/tendinopathy, subacromial impingement, and lateral epicondylitis (28–30). Despite the similar pattern of disease, there is limited evidence on the use of ESWT for medial epicondylitis patients (31–35). Furthermore, at the best knowledge of the authors, no previous research has investigated the beneficial effects of ESWT for medial epicondylitis with concomitant ulnar nerve instability.

Therefore, this study aims to evaluate the effects of ESWT on pain, symptom severity, and functional outcomes of a combined ESWT protocol in patients with medial epicondylitis concomitant to ulnar nerve instability over a minimum of 2 years follow-up.

## 2 Materials and methods

### 2.1 Subjects and clinical assessment

This retrospective study included all patients confirmed as medial epicondylitis with ulnar nerve involvement through medical history and physical examination by ultrasound and who underwent a combined ESWT protocol from September 2019 to May 2023 by the same physician at the Center for Rehabilitative Medicine “Sport and Anatomy” of the University of Pisa. Main search terms used to perform medical chart review were “medial epicondylitis”, “medial epicondyle tendinopathy”, “epitrochleitis”, “ulnar nerve dislocation”, “ulnar nerve instability”, and/or “ulnar subluxation”. The analysis was conducted by two authors who extracted characteristics, treatment measures, and functional outcomes of the patients. Inclusion criteria were: age over 18 years, receiving a diagnosis of medial epicondylitis, concomitant ulnar nerve instability/subluxation, having no previous treatment for medial epicondylitis, and having no corticosteroid injection therapies for any reason during the past month. Subjects were excluded if they had a history of elbow injury or upper limb fractures/surgery, or cervical radiculopathy. At physical examination, patients endorsed pain over the medial elbow that increased on palpation of the medial epicondyle, tenderness over the medial epicondyle, 5–10 mm distally, and resisted wrist flexion with extended elbow. The study population consisted of patients with varying symptom durations, as detailed in Table 1. Clinical assessment of medial epicondylitis was confirmed by US examination by a radiologist (F.B.) with over 20 years of experience in musculoskeletal ultrasound (Figure 1A). Clinical examination with dynamic US was also performed to assess ulnar nerve dislocation during elbow movement from full extension to full flexion (Figure 1B). Remarkably, dynamic US was chosen as it allows real-time, high-resolution visualization of ulnar nerve behavior, and is considered the gold standard method for assessing its stability within the retroepicondylar groove (36). During dynamic evaluation, participants were asked to extend their elbows with forearm supination, then flex gradually until they reached the full flexion position. All examinations were performed by a single board-certified radiologist (F.B.) with extensive experience in ultrasonographic examination of musculoskeletal disorders. Particular care was taken by the examiner to avoid too much pressure on the transducer, as this may cause deformation of the ulnar nerve and prevent its displacement. For diagnostic purposes, the ulnar nerve position at full flexion of the elbow joint was classified into three categories: non-dislocation (nerve remaining within the retroepicondylar groove); partial dislocation (nerve moving onto the tip of the medial epicondyle); and complete dislocation (nerve moving anteriorly beyond the tip of the medial epicondyle). The present positional criterion served as an objective and reproducible diagnostic threshold for participant inclusion. Only patients demonstrating complete nerve dislocation at full elbow flexion were classified as having nerve instability and included in the study. The study was conducted in accordance with local legislation and institutional requirements, and informed consent was obtained from all participants.

**Table 1:** Demographic and anthropometric characteristics of study participants.

Characteristic	Case no. 1	Case no. 2	Case no. 3	No. (%)
Age (years)	56	47	25	
Gender *n* (%)				
Male	1	1	1	3 (100%)
Female	-	-	-	-
BMI (kg/m^2^)	23.6	21.3	23.4	
Dominant hand *n* (%)				
Left	-	-	-	-
Right	1	1	1	3 (100%)
Affected side *n* (%)				
Dominant hand	-	1	1	2 (66.7%)
Nondominant hand	1	-	-	1 (33.3%)
Symptom duration *n* (%)				
0–3 months	-	-	1	1 (33.3%)
3–6 months	1	1	-	2 (66.7%)
6–12 months	-	-	-	-
Repetitive and/or forceful work *n* (%)				
Yes	-	1	1	2 (66.7%)
No	1	-	-	1 (33.3%)
Leisure/sport activities showing repetitive activities *n* (%)				
Yes	1	1	1	3 (100%)^a^
No	-	-	-	-
Frequency of exercising				
<2x/week	-	-	-	-
2–3x/week	1	1	-	2 (66.7%)
>3x/week	-	-	1	1 (33.3%)

^a^ Activity associated with onset of symptoms: canoeing, tennis (serving), biking.

### 2.2 ESWT protocol

The patients underwent a cycle of combined ESWT using a Duolith SD1 (Storz Medical AG., Tägerwilen, Switzerland). A total of three to five sessions were administered at weekly intervals.

The total number of ESWT sessions was selected in accordance with previously published protocols for epicondylitis, in which 3–5 sessions are most commonly reported and associated with significant improvements in pain and functional outcomes (37–39).

The treatment consisted of a sequential application of focal (fESWT) and radial (rESWT) shockwaves for each therapeutic session. fESWT (0.15–0.20 mJ/mm^2^, 5–6 Hz, 1,000 shocks) was performed primarily targeting the medial epicondyle, while rESWT (1.3–1.8 mJ/mm^2^, 14 Hz, 2,000 shocks) was delivered on the myotendinous junction of the flexor-pronator muscle group and the antebrachial fascia. No local anesthesia was applied. The treatment was performed under ultrasound guidance and careful surface anatomy assessment, which allowed clear identification of the ulnar nerve course and ensured that the focal application was delivered at a safe distance from the nerve sheath. Furthermore, the procedure was carried out by an operator experienced in anatomy and musculoskeletal ultrasound, which further minimized the risk of inadvertent nerve exposure. No clinical signs or symptoms of ulnar nerve irritation or injury were observed during or after treatment.

Patients were instructed to avoid the use of non-steroidal anti-inflammatory drugs during the treatment and discouraged from performing pain-triggering activities during the entire cycle of treatment.

### 2.3 Outcome measures

The assessments were performed at baseline (T0), at 2 months post-treatment (T1), and at more than 2 years post-treatment (specifically 35, 37 and 47 months) (T2). The following subjective and functional outcomes were considered:

Reported pain severity, assessed with the 11-point numerical rating scale (NRS), scoring between 0 (no pain) to 10 (worst possible pain);
Upper limb disability, assessed with the Quick-Disabilities of the Arm, Shoulder and Hand questionnaire (Q-DASH). This latter asks about the symptoms as well as the patient's ability to perform certain activities, and it is scored from 0 (best function, no limitation) to 100 (worst function, full disability). The Q-DASH is a shortened version of the self-administered DASH questionnaire, and it contains only 11 items instead of the 30-item DASH outcome measure (40);
Assessment of perceived symptom severity of ulnar neuropathy at the elbow (UNE) through a self-administered questionnaire (UNEQ). In particular, the UNEQ is composed of nine questions, and it considers symptoms (numbness and tingling) of the fourth and fifth fingers, elbow pain, and changes in these symptoms in relation to elbow position. A score from 1 (absence of symptom) to 5 (most severe) is assigned for each question, and the overall score is calculated as the mean of the nine scores (41);
Self-perceived overall pain and activity limitation with the Roles and Maudsley score (R&M), a four-point subjective rating scale that is widely used to assess patients' levels of satisfaction regarding the results of ESWT (42).

## 3 Results

**Figure 2:**
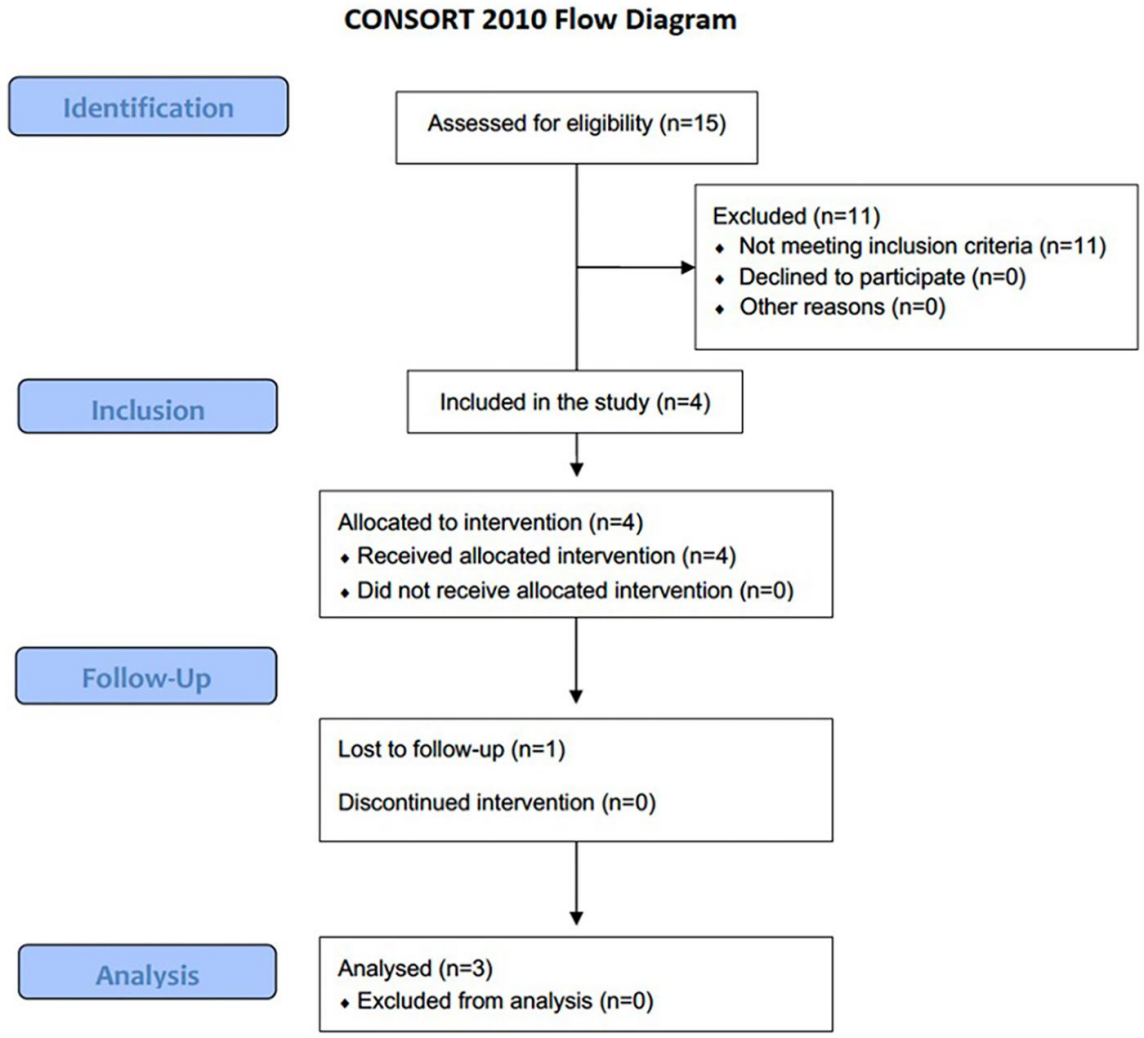
Flow diagram of participant recruitment, allocation, and analysis.

A chart review from two providers identified 15 consecutive patients (14 males and one female) with US-confirmed elbow tendinopathy who were treated with combined ESWT during the study period. Fourteen patients had unilateral medial epicondylitis, and one had bilateral medial epicondylitis. Eleven subjects were excluded as dynamic US did not confirm the occurrence of concomitant ulnar nerve instability. Of the four eligible patients, one subject was excluded from the final analysis because he could not be reached during the follow-up. Thus, only three patients (3 elbows) were included in the final analysis (Figure 2). The descriptive data of study participants are reported in Table 1.

With reference to the ESWT protocol, the number of sessions was predefined to range from a minimum of three to a maximum of five. Importantly, the total number of treatment sessions was adapted based on the individual patients' feedback regarding improvement in symptoms (particularly pain). Two patients (Case no.1 and no. 2) received five treatment sessions, whereas one patient (Case no. 3) underwent three sessions. Regarding the last patient, the therapy was discontinued due to the absence of perceived benefit and lack of patient's reported satisfaction with ESWT treatment.

As reported in Table 2, two months after treatment, two patients had lower NRS scores (by 5–7 points) than before ESWT. Even at long-term follow-up, these patients continued to experience a noticeable reduction of pain (by 6–8 points). By the final follow-up, one patient was completely pain-free. Compared to the pretreatment condition, the Q-DASH and UNEQ scores decreased markedly over time in these two patients, who were satisfied with the outcome, as evidenced by the R&M scores. In one patient, all outcomes remained unchanged at all follow-up time points, and the treatment did not result in any noticeable improvement or decrease in symptoms. No patient exhibited complications during the treatment, and no side effects were reported.

**Table 2:** Clinical outcomes at each time point of the study.

Outcome measure	Case no. 1	Case no. 2	Case no. 3
NRS			
T0	7	8	8
T1	2	1	8
T2^a^	1	0	7
Q-DASH			
T0	52.3	50	36.4
T1	4.5	9.1	34.1
T2^a^	0	0	31.8
UNEQ			
T0	2.33	2.67	3.33
T1	1.11	1	4
T2^a^	1.11	1	3.44
R&M			
T0	4	4	4
T1	3	3	4
T2^a^	1	1	4

NRS, numerical rating scale; Q-DASH, Quick-Disabilities of the Arm, Shoulder and Hand questionnaire; UNEQ, ulnar neuropathy at the elbow questionnaire; R&M, Roles and Maudsley score.

^a^ Case no. 1, = 47 months; Case no. 2 = 37 months; Case no. 3 = 35 months.

## 4 Discussion

Medial epicondylitis is a degenerative overuse painful syndrome involving the common flexor-pronator tendon of the upper limb. Even though less reported than lateral epicondylitis, medial epicondylitis can be an equally troublesome and debilitating condition affecting both athletes and the general population. Due to the low incidence of medial epicondylitis and the limited number of clinical studies on conservative treatments for this condition, no standard protocol has currently been established. Most studies on ESWT treatment for elbow pain concern lateral epicondylitis (18, 29, 30). In contrast, there is a paucity of existing literature on the use of ESWT in medial epicondylitis with somewhat heterogeneous results. This, in turn, is mainly due to methodological heterogeneity between studies regarding shockwave application, such as the number of treatment sessions, energy doses, and types of shockwave generators, making it difficult to compare clinical results or draw firm conclusions (31–35). Additionally, because of the complexity of the elbow region, appropriate management of medial epicondylitis patients depends on a thorough understanding of medial elbow anatomy and an accurate diagnosis that considers other related or co-occurring pathologies that result in medial elbow pain. Above all, ulnar nerve involvement, which is frequently described in medial epicondylitis patients.

To our knowledge, this study is the first retrospective research to explore the safety and the effect of ESWT in medial epicondylitis patients with concomitant ulnar nerve instability. In detail, in the present study, we adopted a combined sequential protocol using both radial and focal shock waves, based on the complementary biological and physical effects of the two modalities. Radial shock waves were applied first to promote increased local perfusion and blood recruitment in the inflamed peri-tendinous tissues, thereby enhancing the local biological environment and supporting reparative processes (43). In addition, radial shock waves were also applied over the antebrachial fascia to exert a mechanical, massage-like effect on the fascial tissue, aiming to improve tissue mobility and reduce myofascial tension. Subsequently, focal shock waves were used to reach deeper tissues and precisely target the primary site of pathology at the medial epicondyle, where controlled high-energy delivery is required to act directly on the damaged tendon insertion (43, 44). This sequential approach was therefore chosen to combine a global biological stimulation of superficial tissues with a targeted mechanical and biological effect at the deeper pathological focus, which would not be achievable with a single modality alone.

In particular, the results of this study showed that ESWT has promising long-term effects on pain relief and functional improvement in the majority of patients. However, the fact that one patient showed no change in outcome scores over the entire study raises some clinical concerns. Although it cannot be conclusively determined from the present study, the lack of clinical improvements observed in this patient may be influenced by a combination of patient-specific factors. In particular, it is possible that the patient's symptoms were related to ulnar nerve instability, potentially due to congenital anatomical factors, such as a shallow bony retroepicondylar groove and/or a loose Osborne's ligament, rather than a nerve irritation caused by tendinopathy-related regional inflammation around the elbow. The poor clinical results following shockwave therapy in this patient may have been more likely due to this underlying anatomical issue, as ESWT may have provided only minimal benefit on tendon pain, while being unable to address the underlying mechanical instability of the nerve. In addition to the anatomical factor, the patient also presented with the shortest symptom duration, suggesting a possible acute phase of tendinopathy. Compared to chronic degenerative tendinopathies, there is limited evidence on the effectiveness of ESWT in acute tendinopathies. This, in turn, could have contributed, at least in part, to the patient's minimal response to treatment. Finally, the absence of perceived clinical improvement led to the early discontinuation of ESWT treatment, which may have further limited its therapeutic potential, highlighting the importance of monitoring patient response early in the treatment process. Despite the speculative nature of the above hypotheses, it remains clear that this patient did not respond to ESWT therapy, which is an important finding that warrants further investigation in larger cohorts to identify predictive factors for non-response. Future studies should aim to investigate specific characteristics and factors influencing the outcome of ESWT that could predict non-response to ESWT, such as acute symptom onset, patient age, and underlying anatomical factors. This, in turn, would help refine treatment protocols, improve patient selection, and ensure more personalized and targeted approaches for management of tendinopathy.

Despite this, in any case, no treatment-related complications or adverse effects were encountered during the entire follow-up period, which reinforces the overall safety profile of ESWT and is also in line with previous literature (31, 33, 35).

As for other tendinopathies, most of the biological responses induced by ESWT on tendon tissue rely on accelerating the metabolism of inflammatory mediators, promoting neovascularization and tendon repair mechanisms, as well as inhibiting pain signaling, which are very useful in clinical settings (21, 45). As another hypothesis, the mechanism of action is that, in addition to its direct effect on tendon tissue, ESWT could also suppress inflammatory responses while improving nerve tissue blood flow and perfusion, which potentially contributes to symptom relief in peripheral neuropathy (46).

Although the biological plausibility of the results, further research is needed to fully understand the molecular mechanisms underlying the beneficial effects of ESWT in musculoskeletal conditions like medial epicondylitis.

## 5 Conclusions

While less prevalent than its lateral counterpart, medial elbow tendinopathy presents diagnostic and therapeutic challenges, particularly in cases with coexisting ulnar nerve involvement, as dynamic ulnar nerve instability may exacerbate tendinopathy symptoms and complicate its management. Understanding the anatomo-pathological interplay between the mechanical tendon degeneration, nerve irritation, and instability is crucial for optimizing diagnosis and tailoring treatment strategies. Nonetheless, further studies are warranted to clarify the pathophysiological mechanisms and guide evidence-based intervention for this overlooked condition.

The limitations of this study are those inherent to retrospective designs and the small sample size. In particular, the inclusion of only three patients significantly limits the external validity of the present findings, which should be interpreted with caution. Further studies with larger sample sizes and prospective designs are needed to confirm these findings and enhance the generalizability of the conclusions.

Nevertheless, to the best of our knowledge, it represents the first evaluation of long-term outcomes regarding pain relief and functional improvement in patients with medial epicondylitis complicated by ulnar nerve involvement who were treated with ESWT. While these findings may offer novel insights into the safety and potential effectiveness of ESWT for upper limb tendinopathy, further prospective studies with larger cohorts are needed to establish the optimal treatment protocol for this condition.

## Data Availability

The raw data supporting the conclusions of this article will be made available by the authors, without undue reservation.
